# Mutual-Information-Based Incremental Relaying Communications for Wireless Biomedical Implant Systems

**DOI:** 10.3390/s18020515

**Published:** 2018-02-08

**Authors:** Yangzhe Liao, Mark S. Leeson, Qing Cai, Qingsong Ai, Quan Liu

**Affiliations:** 1 School of Information Engineering, Wuhan University of Technology, Wuhan 430070, China; yangzhe.liao@whut.edu.cn (Y.L.); qingsongai@whut.edu.cn (Q.A.); quanliu@whut.edu.cn (Q.L.); 2 Key Laboratory of Fiber Optic Sensing Technology and Information Processing, Wuhan University of Technology, Ministry of Education, Wuhan 430070, China; 3 School of Engineering, University of Warwick, Coventry CV4 7AL, UK; mark.leeson@warwick.ac.uk

**Keywords:** WBANs, communication protocol, QoS, network lifetime

## Abstract

Network lifetime maximization of wireless biomedical implant systems is one of the major research challenges of wireless body area networks (WBANs). In this paper, a mutual information (MI)-based incremental relaying communication protocol is presented where several on-body relay nodes and one coordinator are attached to the clothes of a patient. Firstly, a comprehensive analysis of a system model is investigated in terms of channel path loss, energy consumption, and the outage probability from the network perspective. Secondly, only when the MI value becomes smaller than the predetermined threshold is data transmission allowed. The communication path selection can be either from the implanted sensor to the on-body relay then forwards to the coordinator or from the implanted sensor to the coordinator directly, depending on the communication distance. Moreover, mathematical models of quality of service (QoS) metrics are derived along with the related subjective functions. The results show that the MI-based incremental relaying technique achieves better performance in comparison to our previous proposed protocol techniques regarding several selected performance metrics. The outcome of this paper can be applied to intra-body continuous physiological signal monitoring, artificial biofeedback-oriented WBANs, and telemedicine system design.

## 1. Introduction

Wireless body area networks (WBANs) are becoming increasingly popular recently due to their suitability and flexibility in medical and nonmedical applications. WBANs deploy low-power biosensors and devices inside, on, or around the human body [1,2]. The biosensors are able to sense and transmit real-time physiological and contextual information profiling human body activities such as rehabilitation monitoring, blood pressure, and digital medical treatment. Even more fascinating and promising is the near-future healthcare revolution where biomedical devices implanted inside the human body are wirelessly connected and organized for early disease detection or organ transplantation monitoring over an extended period [3]. Data transmission can be conducted between implanted devices and an external coordinator, which is furnished with high computational capacity, sufficient memory space, and an appropriate power source. Often in modern healthcare, the coordination function may be performed by a contemporary smartphone. Intra-body implanted sensor networking is expected to bring revolutionary health applications and services. However, there exist several technical constraints in wireless in-body sensor networks. Analysis of intra-body communication channel characteristics is a crucial research challenge due to the complex in-body transmission environment. Radio frequency signals suffer significant energy attenuation when transmitting from the intra-body area to the on-body region [3,4]. Moreover, owing to the strict technical constraints of implanted device battery design, the limited energy supply is also a major bottleneck for a wireless in-body sensor network [1,5]. 

Since the network energy consumption is directly related to the overall communication distance, relay-based routing protocol techniques are considered to reduce the transmission distance [6]. Relay-based energy efficient protocol routing enables the selection of the best possible data transmission route, which significantly decreases the transmission length. Moreover, cooperative communications have been applied to the protocol design to address research difficulties related to prolonging the network lifetime and improving the energy efficiency [7]. We consider using implantable devices employed with biosensors that transmit their sensed physiological data to a coordinator through wearable devices which perform as on-body relays. By forwarding the sensed data to the relays, the complexity and power consumption are transferred from the implanted sensor to the relay node, which may easily be recharged. 

According to [8,9], for continuous healthcare monitoring services, not all sensed data needs to be transmitted to the external coordinator. For example, when the current scanned information is similar to the previously sent message where both of them are within the normal range, transmission would be highly redundant. Therefore, the motivation to improve the lifetime of all implanted sensors is to reduce the amount of redundant normal data transmission. In addition, data transmission from different implanted sensors is affected by noise and channel fading, which result in data packet loss and increased path loss. The coordinator can gather the received information packets from relay nodes and implanted sensors using numerous data combining techniques, which are energy efficient schemes to enhance the network reliability in the presence of channel fading. Moreover, this paper is also motived by the tradeoff between the transmitting power and various quality of service (QoS) metrics for implanted sensors, considering the power constraints and the essential network reliability regarding the outage probability (OP). 

In this paper, we present an MI-based incremental cooperative routing protocol for wireless in-body sensor networks. The primary target of the proposed protocol is to maximize the network lifetime. Firstly, MI criteria are considered to prevent redundant information transmission. Moreover, by adopting an incremental cooperative relay-based routing scheme, the energy consumption of the implanted sensors is significantly reduced because the overall communication distance is minimized. We allow the implanted sensor to establish a path directly to the coordinator or to employ a relay node, depending on the transmission distance. Also, a list of selected QoS metrics have been derived via linear mathematical modeling along with the related subjective functions where the amplify and forward (AF) technique and fixed ratio combining scheme are utilized at the relay node and the coordinator, respectively. 

The rest of the paper is organized as follows: Section 2 introduces related work in WBAN routing protocol design. Section 3 summarizes the system architecture and numerous basic models. The proposed communication protocol and the analysis of the relevant QoS metrics are given in Section 4 and Section 5, respectively. Performance evaluation and comparison with our previous work are illustrated in Section 6, and Section 7 concludes the paper.

## 2. Related Work

Typically, WBANs are categorized into wearable and implanted communication networks. Wearable sensor networks offer data transmission between a data sink and a collection of wearable devices, and have been widely accepted in modern healthcare monitoring services such as electromyography (EEG) and body temperature. Unlike wearable devices, in-body sensors are implanted inside the human body by surgery and, thus, it is difficult to replace them. The maximization of the implanted sensor’s lifetime is an emerging area of research in WBANs [1,10,11,12,13]. However, the majority of recent studies have focused on wireless wearable sensor networks and very few have been proposed for wireless in-body sensor networks. 

The authors in [14] proposed a prototype for heterogeneous body sensor networks. The placement of different types of sensor nodes is based on their data rate; a live sensor node with a high data rate enables direct communication to the sink and forwards data received from low-data-rate sensors. The results show that the network consumes less energy as compared with multi-hop-based transmission.

A series of routing techniques choose body temperature as a primary factor. In [15], the authors proposed a thermal-aware QoS routing method, which employs a localized approach in communication route selection. The work in [16] reported a routing scheme known as TARA, based on localized temperature; the sensor node with the minimum temperature value is selected as the forwarder during the data transmission. This kind of wireless network enables the heat generated by the implanted sensors caused by radiation and circuitry power consumption to be balanced.

The authors in [17] proposed an augmented efficiency global routing scheme. The proposed routing protocol considered a dynamic energy cost technique that limits power expenditure among all body sensors to the minimum level possible. This results in reduced energy consumption per bit and brings an extensive improvement of up to 40% to the network lifetime. 

Hybrid protocol approaches have been described as an efficient way to improve the network energy efficiency. In [18], the authors combined two forms of data transmission modes—single-hop and multi-hop—with their protocol design. In detail, the one-hop scheme is only applied to emergency data transmission while the multi-hop technique is for normal data communication. The results show that this method achieves more reliable message transmission as compared with pure multi-hop communication. 

Relay-based communication is reported as an efficient way to prolong the network lifetime. In [19], the authors proposed an incremental-relay-based routing protocol and compared it with the traditional two-relay-based protocol. The results show that incremental relaying protocol significantly decreases the network energy consumption for the reason that the second relay node only activates and forwards sensed data from the in-body sensor to an external coordinator after the first relay node fails.

Cooperative transmission is an efficient way to enhance system performance since it allows all sensor nodes to share resources within a network. In [20], the authors reported that their cooperative routing technique offers satisfactory performance regarding numerous QoS factors coupled with low energy consumption. This is achieved by applying a competitive mechanism to all sensor nodes by operating a multi-agent reinforcement learning algorithm. Additionally, in [21,22], the results demonstrated that the cooperative scheme provides better performance than single-link transmission under a mobile, dynamic condition in terms of co-channel interference mitigation and the OP.

## 3. System Model

### 3.1. Network Model

**Figure 1:**
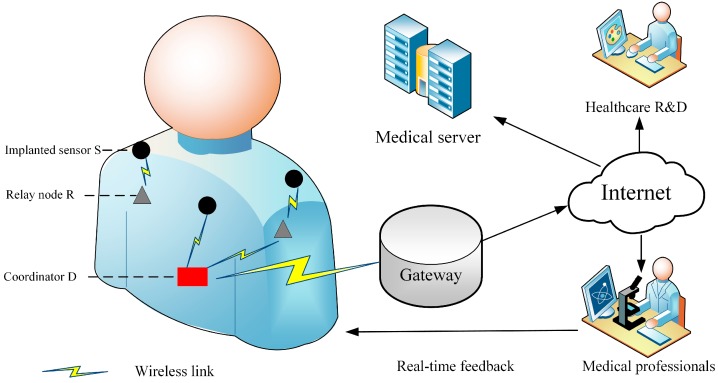
The configuration of wireless biomedical implant networks.

A wireless biomedical implant system model is shown in Figure 1 and consists of a series of implanted sensors, on-body relay nodes, and the coordinator [1,2]. A WBAN deploys a star topology and has only one coordinator. Thanks to the rapid progress of the medical Internet of Things, the coordinator can be connected to the Internet via the gateway and the collected data can be stored on the medical server and prepared for future R&D. In addition, abnormal data can be provided to medical professionals who are able to offer emergency feedback to the patient promptly. Detailed information of the system model is given as follows [1]:

- Implanted sensor (S): A device that is implanted by surgery inside the human body. A deep tissue implant is positioned at a distance up to 9 cm below the skin.
- On-body relay node (R): This is considered to be a device that is located on the body surface or up to 2 cm from it. R is usually selected as a wearable computing device and is capable of forwarding the gathered data received from implanted devices to the coordinator.
- Coordinator (D): An access point, placed either on human clothes or close to the human body. A smartphone or other personal digital device is usually selected to act as a coordinator.

The communication paths between the *i*th implanted sensor and the *j*th on-body relay node are represented as $S_{i}R_{j}$, where i∈{1,2,…n} and j∈{c,ω}. It is worth noting that the implanted sensor node ID is assigned by the coordinator and j is the location of the on-body relay node on the human body, in accordance with [23], and c and ω mean the chest and wrist, respectively. Similarly, the paths between the on-body relay node and the coordinator are denoted $R_{j}D$ and the links between the implanted sensor and the coordinator are represented as $S_{i}D$.

### 3.2. Path Loss Model

Due to the naturally lossy intra-body environment, the data transmission from the in-body region to the on-body area suffers considerable power attenuation [1]. Moreover, the positions of the on-body relay nodes and the coordinator affect the communication link quality and transmission distance. The path loss (PL) model can be formulated as a function of the distance *d* between an implanted sensor to the on-body device [10]:

(1) $$PL_{dB}\left(d\right)=PL_{dB}\left(d_{ref}\right)+10n\log_{10}\left(\frac{d}{d_{ref}}\right)+s_{dB},d\geq d_{ref}$$

where $d_{ref}$ is the reference distance; $PL_{dB}\left(d_{ref}\right)$ denotes the corresponding PL value; and *n* and $s_{dB}$ represent the PL exponent and the shadow fading parameter, respectively.

### 3.3. Energy Consumption Model

To date, numerous radio models have been published in the literature. In this paper, the energy consumption analysis of the wireless implant biomedical network is employed by extending our flexible QoS WBANs model published in [5]. The transmission energy consumption means the power consumed while sending the sensed packets and the associated control overhead on the radio. Assuming the length of the data packet is *k* bits, the minimal transmission energy consumption of the implanted sensor $E_{Tx\_min}$ during any time period can be defined as

(2) $$E_{Tx\_min}\left(d,k\right)=kE_{Tx_{elec}}+kE_{amp}dn$$

where *n* represents the intra-body PL exponent as mentioned in Equation (1) and $E_{amp}$ is the radio amplifier energy consumption. $E_{Tx\_elec}$ denotes the essential power consumption to activate the transmitter electronic circuit per bit. Similarly, the minimal energy consumption of the receiver $E_{Rx}\left(k\right)$ can be expressed as $E_{Rx}\left(k\right)=kE_{Rx\_elec}$, where $E_{Rx\_elec}$ is the essential power consumption to activate the receiver electronic circuit per bit. The nRF2401A is a commercially available and low-power transceiver in WBANs at 2.4 GHz, and has been adopted in numerous research outputs [10,11,24]. We thus deploy the nRF2401A energy consumption parameters for further simulation analysis.

### 3.4. Mutual Information Model 

As reported in [24,25,26], continuous intra-body normal data transmission results in significant power wastage and reduces the network lifetime. This is because scanned information is similar or close to the previously sent data and does not contain any emergency medical information. In this paper, we adopt a mutual information approach to minimize redundant normal range data transmission. The MI, I(X;Y), can be regarded as the amount of uncertainty in *X* due to the knowledge of *Y*. The expression for MI can be defined as

(3) $$I\left(X;Y\right)=\underset{x,y}{\sum }p\left(x,y\right)\log\frac{p\left(x,y\right)}{p\left(x\right)p\left(y\right)}$$

where p(x) and p(y) are the probability distribution functions of *X* and *Y*, respectively. Let $D_{x}$ and $D_{y}$ represent the scanned data set of an in-body sensor node at the continuous time slots $t_{x}$ and $t_{y}$, the mutual information then can be rewritten as

(4) $$I\left(D_{x};D_{y}\right)=\underset{x}{\sum }\underset{y}{\sum }p\left(D_{x},D_{y}\right)\log\left(\frac{p\left(D_{x},D_{y}\right)}{p\left(D_{x}\right)p\left(D_{y}\right)}\right)$$

assuming that $D_{x}$ is within its normal range. According to [25], a higher value of $I\left(D_{i};D_{j}\right)$ implies that during continuous time slots $t_{x}$ and $t_{y}$ (the next time slot), the in-body device senses similar or the same physiological information. The collected data packet $D_{j}$ is not allowed to transmit if found to be similar to $D_{x}$. By this means, the implanted sensor can avoid redundant continuous data transmission and therefore decrease its total energy consumption. 

### 3.5. Outage Probability (OP)

This is an important performance metric for measuring the network reliability. Detailed information concerning OP can be found in [27,28]. The expression of OP under the single-relay condition can be expressed as

(5) $$ℛ=\frac{1}{2}\log_{2}\left(1+{\left|h\right|}^{2}\frac{P_{t}}{N_{0}}\right)$$

where $P_{t}$ is the transmitting power and $N_{0}$ denotes the noise power; *h* represents the channel gain of the intra-body fading channel; and ${ℛ}_{SR}$ and ${ℛ}_{RD}$ denote the mutual information of the SR and RD communication links. The maximum mutual information of the SD communication path should be followed by ${ℛ}_{SD}=min\left\{{ℛ}_{SR},{ℛ}_{RD}\right\}$. The OP of the communication link between the in-body sensor to an on-body relay node for a given data rate γ can be written as $P_{SR_{i}}=P\left\{min\left\{{ℛ}_{SR_{j}},{ℛ}_{R_{j}D}\right\}<\gamma \right\}$. The intra-body PL model follows a lognormal distribution at stated in Section 3.2; thus, ${\left|h\right|}^{2}$ follows a lognormal distribution and the maximum mutual information can be written as

(6) $$P\left\{ℛ<\gamma \right\}=\frac{1}{2}erfc\left[-\frac{\ln\left(\left(2^{2\gamma }-1\right)N_{0})/P_{t}\right)-\mu }{\sqrt{2}\sigma }\right]$$

where erfc(.) denotes the complementary error function [27]. According to [10], the in-to-out body PL model parameters are such that μ = 0 and σ = 2.93. Equation (6) can be rewritten as $P\left\{ℛ<\gamma \right\}=\frac{1}{2}erfc\left[-\frac{\ln\left(\left(2^{2\gamma }-1\right)N_{0})/P_{t}\right)}{4.14}\right]$. Considering a wireless biomedical implant network with two on-body relay nodes, each of the relay nodes performs an AF operation on the information packets it receives from the implanted sensors and sends to the coordinator. The maximum mutual information ${ℛ}_{SD}$ can be expressed as

(7) $${ℛ}_{SD}=\overset{2}{\underset{j=1}{\prod }}\left[1-\left(1-P\left\{{ℛ}_{SR_{j}}<\gamma \right\}\right)\left(1-P\left\{{ℛ}_{R_{j}D}<\gamma \right\}\right)\right]$$

where ${ℛ}_{SD}$ should also be subject to ${ℛ}_{SD}=min\left\{{ℛ}_{SR_{j}},{ℛ}_{R_{j}D}\right\},\;\forall j\in \left\{1,2\right\}$.

### 3.6. Data Combining Scheme

Demonstration of the cooperative routing process can be found in [6,7]. In the first phase, the implanted sensor transmits its sensed data to both the relay node *R* and the coordinator *D*, simultaneously. The information sent from *S* to *R* and from *S* to *D* in this first phase can be given as

(8) $$y_{SR}=S_{SR}\cdot \left(PL_{dB}\left(d_{ref}\right)+10n\log_{10}\left(\frac{d_{SR}}{d_{ref}}\right)+s_{dB}\right),$$

(9) $$y_{SD}=S_{SD}\cdot \left(PL_{dB}\left(d_{ref}\right)+10n\log_{10}\left(\frac{d_{SR}+d_{RD}}{d_{ref}}\right)+s_{dB}\right),$$

where $S_{SR}$ and $S_{SD}$ represent the slow fading effect that is multiplicative in the intra-body environment. Then, *R* forwards the received data to *D*; similar to Equations (8) and (9), the total information received at *D* can be expressed as

(10) $$y_{RD}=f\left(y_{SR}\right)\cdot \left(PL_{dB}\left(d_{ref}\right)+10n\log_{10}\left(\frac{d_{RD}}{d_{ref}}\right)+s_{dB}\right)$$

where $f\left(y_{SR}\right)$ represents the processing functions applying on the received signal from *S* to *R*. It should be noted that some information packets are dropped during the transmission process due to the transmission channel fading effect. The coordinator then utilizes a diversity combining scheme to gather the received data from the implanted sensors and the relay nodes. 

## 4. The Proposed Protocol

In this section, a novel communication protocol for wireless biomedical implanted networks is demonstrated. Considering the limited battery of the implanted sensor and that every piece of abnormal medical data information is potentially life-critical, we aim to prolong the network lifetime and maintain the communication link quality. A communication link can be established only when the mutual information value $I\left(D_{i};D_{j}\right)$ value becomes smaller than the predetermined threshold. The communication direction can be either from *S* to *D* or from *S* to *R*; then *R* forwards the data collected to *D*, depending on the transmission distance.

### 4.1. Network Initialization Stage

The network starts to monitor the patient’s health status. In accordance with [23], once network initialization is finished, the coordinator broadcasts an information message, which informs all body sensors of its location. Moreover, all implanted sensors are assigned with unique IDs and notified with the relay nodes’ positions by the coordinator. Then, all implanted sensors store the locations and broadcast information packets consisting of the location, energy status, and IDs to the relay nodes. In such a manner, all implanted sensors can give updates of their positions and energy status information to the relay nodes and the coordinator.

### 4.2. Transmission Route Selection

#### 4.2.1. Transmission Route Selection

**Figure 2:**
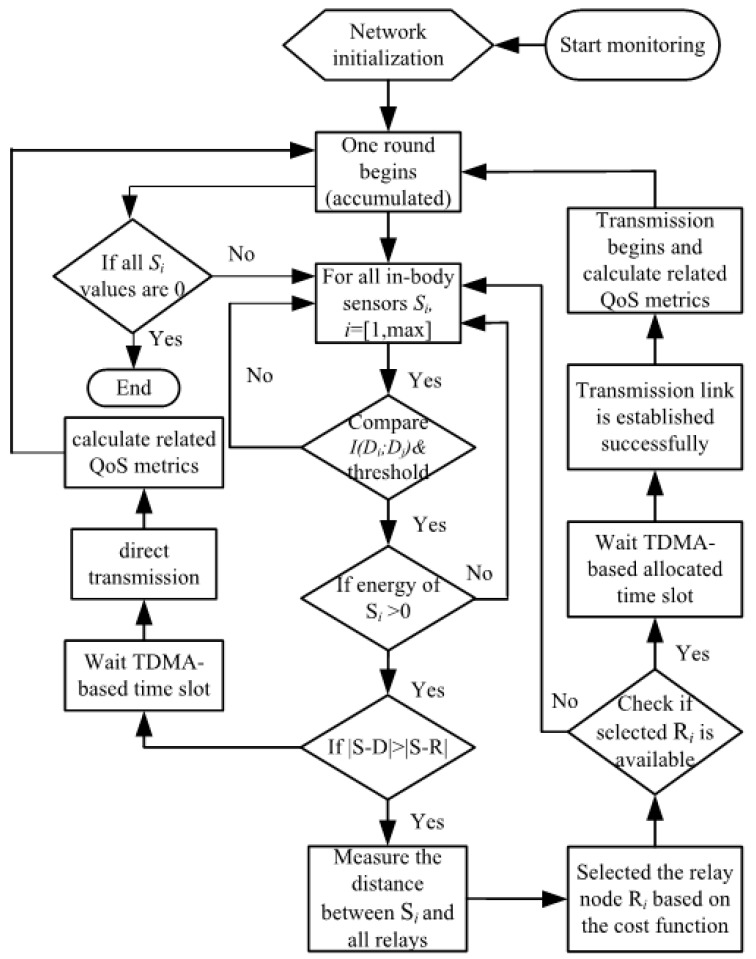
The communication flow of the proposed protocol.

The communication flow of the proposed protocol is given in Figure 2. Since the relay nodes and the coordinator are attached to clothes, the implanted sensor is able to transmit the sensed data either to the relay node or the coordinator directly. As demonstrated in Equation (1), longer communication distances suffer higher energy consumption. Thus, if the transmission implant sensor *S* is closer to *R*, it utilizes the cooperative transmission scheme. The transmission scheme selection function c(i) is given as

(11) $$d\left(i\right)=\{\begin{array}{cc} d_{SR_{i}}>d_{R_{i}D}, & \text{direct transmission}\\ d_{SR_{i}}\leq d_{R_{i}D}, & \text{relay cooperative transmission} \end{array}$$

where when $d_{SR_{i}}>d_{R_{i}D}$, the transmission link is established directly between the implanted sensor and the coordinator. The coordinator allocates time division multiple access (TDMA)-based time slots to the in-body sensors to avoid data collision [19]. A series of QoS metrics will be calculated during the data transmission and then a new round will start once the transmission is finished. Otherwise, the communication link is created between the implant sensor and the corresponding relay node. Detailed information on the relaying transmission approach is illustrated in Section 3.2 to Section 3.4.

#### 4.2.2. Cooperative Technique 

When $d_{SR_{i}}\leq d_{R_{i}D}$, the relay-based cooperative technique is selected for the data transmission. The communication flow is shown as follows: implant sensor → body relay → coordinator. However, it is clear that if there are numerous relay nodes in the network, the implanted sensor may have several available communication links to deliver sensed data. To balance energy consumption among all implanted sensors, the proposed protocol chooses a new relay node in each round. The relay selection algorithm is given as [29]

(12) $$C\left(i\right)=\frac{d\left(i\right)}{E\left(i\right)}$$

where $i\in \left\{S_{1},S_{2}\ldots S_{n}\right\}$, d(i) denotes the transmission distance between the in-body sensor node *i* and the potential relay node, and E(i) represents the residual energy of the implanted sensor *i*. The relay node with the minimal cost function value is selected for data transmission.

### 4.3. Data Transmission Stage

At this period, TDMA operates in the communication process to guarantee no interference or data collision during data transmission. The coordinator assigns TDMA-based time slots to the relay nodes and the in-body sensors. The in-body sensor transmits the collected data to the selected relay node in the scheduled period. Then, the relay node forwards the aggregated data to the coordinator as mentioned in Section 3.6.

### 4.4. Data Combining Strategy

Once the corresponding relay node is selected, the received data are transmitted to the coordinator through the implanted sensor and relay node. The collected data combining scheme at the coordinator can be expressed as

(13) $$y_{D}=\alpha y_{SD}+\beta y_{RD}$$

where $y_{D}$ represents the combined output signal at the coordinator; and α and β are the weights of the two communication links *SD* and *RD*, respectively. The ratio α/β reflects the communication link quality and captures the influence of the channel shadowing effect on the transmission channel. The ratio can be formulated as a function of distance when the relay node is selected:

(14) $$\frac{\alpha }{\beta }=\frac{d_{SR_{j}}+d_{R_{j}D}}{d_{R_{j}D}}$$

where α/β is related to the transmission distances $d_{SR_{j}}$ and $d_{R_{j}D}$.

## 5. Analysis of Selected QoS Metrics

The intra-body signal transmission suffers significant energy attenuation. To support stable data transmission and maintain link quality, some key QoS metrics are now selected and analyzed.

### 5.1. Network Lifetime Modeling

Since the implanted sensor is difficult to replace or recharge, it is crucial to maximize the implanted sensors’ lifetime. Two performance metrics—stability period and network lifetime—are chosen for further analysis. The stability period and total network lifetime represent the lifecycle of the wireless biomedical implant systems until the first implanted sensor is energy depleted and all implant sensors’ power is consumed, respectively. The aim of the proposed protocol is to maximize the network lifetime *T*, which is the summation of all rounds:

(15) $$\underset{k,{ℛ}_{SD},d_{SR_{i}},d_{R_{i}D}}{\max}\underset{r}{\sum }t_{r}$$

where *r* and $t_{r}$ describe the current round and the summation of all rounds, respectively.

This is subject to

(16) $$t_{r}\leq \frac{NE_{0}}{\sum_{i}k\left(E_{sen}+E_{pro}+E_{trans}+nE_{amp}d_{SR_{j}}\right)},\;i=1,2,3\ldots ,\;N,$$

(17) $$0\leftarrow E_{0}<E_{Initial},\;i=1,2,3\ldots ,N,$$

(18) $${ℛ}_{SD}=\min\left\{{ℛ}_{SR_{j}},{ℛ}_{R_{j}D}\right\},\;j=1,2,$$

(19) $$\underset{C\left(i\right)}{\min}d_{S_{i}R_{j}},\;i=1,2,3\ldots ,N,\;j=1,2,$$

(20) $$\underset{c\left(i\right)}{\min}d_{R_{j}D},\;j=1,2.$$

As shown in Equation (15), the primary objective target is to maximize the network lifetime *T*. The $E_{sen}$ and $E_{pro}$ represent the energy consumption of sensing per bit and data processing per bit, respectively. The network lifetime is the summation of rounds until all implanted sensors’ energy $NE_{0}$ is depleted. Equation (16) gives the minimal energy consumption for one round. By employing an energy-efficient communication protocol, the energy consumption regarding $E_{trans}$ and $nE_{amp}d_{SR_{i}}$ can be significantly reduced. The function (17) points out that all implanted sensors are provided with the same initial power $E_{Initial}$. Once the network starts to work, the residual energy status of $E_{0}$ steps down and finally reaches zero. Equation (18) illustrates that due to the nature of the communication channel, numerous data packets will be dropped. Equations (19) and (20) demonstrate that the cooperative routing protocol should ensure the transmission distances $d_{SR_{i}}$ and $d_{R_{i}D}$ are kept to the minimal possible values, where path selection schemes C(i) and d(i) are mentioned in Section 4.2.

### 5.2. Network Throughput 

The network throughput denotes the number of successfully received data packets at the coordinator. As stated in Equation (2), transmission of a higher number of data packets demands significant energy consumption. The maximization of the network throughput $y_{D}$ can be expressed as

(21) $$\underset{P_{t},C_{SR_{j}},C_{R_{j}D},\left|N\right|}{\max}y_{D}$$

where $y_{D}=\alpha y_{SD}+\beta y_{RD}$ as mentioned in Equation (14).

This is subject to

(22) $$P>P_{min},$$

(23) $$P_{t}\leq P_{max},$$

(24) $$C_{SR_{j}}=1,\;j=1,2,$$

(25) $$C_{R_{j}D}=1,\;j=1,2,$$

(26) 0<|N|≤i, i=1,2,…,n.

The objective of Equation (21) is to maximize the total number of received packets $y_{D}$ at the coordinator from the implanted sensors and relay nodes during the network lifetime T. To maximize $y_{D}$, the coordinator implements a fixed-ratio combining strategy where the optimal ratio value of 2 for Equation (14) is taken into account in accordance with [29]. Equation (22) demonstrates that the OP should be higher than the minimum required value $P_{min}$ as proved in Equation (6) under the minimum required BER of 10^−3^ [25,30]. According to Equations (5)–(7), higher transmission power $P_{t}$ promises better link quality. However, this causes body temperature rise and may damage the human body; therefore, $P_{t}$ should be carefully designated to guarantee human safety. The constraint in Equation (23) implies that the value of $P_{t}$ should not be more than that of the maximum transmitting power $P_{max}$ as regulated by the IEEE 802.15.6 technical standard [1]. Equation (23) fulfills the condition that all possible transmission paths from *S* to *R* can be established. The constraints in Equations (24) and (25) state that the network should minimize the number of dropped information packets when transmitting from *S* to *R* and *R* to *D*, respectively. Moreover, it is worth noting that the number of transmitted packets from *S* to *R* should be no more than the flow from *R* to *D* as analyzed in Equation (18). Equation (26) offers that the total number of implanted sensors within a WBAN should be limited to reduce the channel access contention and data collision. Since the primary target is to maximize the network lifetime, there exists a tradeoff between network energy consumption and other performance metrics such as throughput, transmission distance, and so forth. One should note that by adopting the optimization of (15), unlike the work in [10], the transmission link is not always applicable to deliver data to the relay or the coordinator, which results in decreasing the number of transmitted packets. Moreover, due to the nature of human mobility and the instability of the intra-body channel, even when using the maximum allowed transmission power, numerous data packets are still dropped or lost during the transmission process. In this paper, we do not consider data retransmission and the computation capacity of wearable relays and the coordinator. To support retransmission of the dropped packets, surplus energy consumption is required and may significantly reduce the network lifetime as we analyzed in (16). Detailed information regarding retransmission can be found in [31,32]. The computation capacity of the relay nodes and the coordinator is not considered. According to [33], computation-intensive or latency-sensitive medical data can be handled locally using edge computing techniques if the wearable device or the coordinator have enough local data storage size and calculation resources, or transfer data to the Internet of Things (IoT) cloud for further computing and analysis.

## 6. Performance Evaluation and Discussion

**Figure 3:**
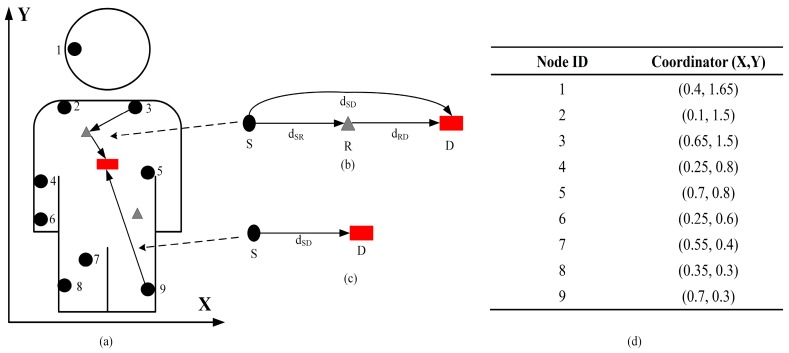
The topology of the proposed protocol. (**a**) Demonstration of the topology; (**b**) relay-based communication; (**c**) direct communication; (**d**) the coordination of implanted sensors.

The topology of the proposed protocol is shown in Figure 3. As shown in Figure 3a, wirelessly networked implanted sensors are placed inside the human body in predetermined locations and monitor physiological parameters such as glucose and insulin levels. The number of implanted sensors is limited to nine on the basis of Equation (26). Figure 3b,c illustrate the relay-based and direct communication, respectively. The coordinates of all implanted sensors (with IDs) are summarized in Figure 3d. The coordinator is fixed at the center of the human body with a coordinate (0.4, 0.85). The implanted sensors transmit the sensed data simultaneously on two data transmission paths via a cooperative scheme to reduce information packet loss. Once the mutual information value I becomes smaller than the predetermined threshold, a cooperative data transmission link is established. Relay nodes are used for cooperative transmission that promises implanted sensors multiple transmission links simultaneously. Moreover, it is worth noting that the data flow from *S* to *R* should be no more than the flow from *R* to *D*. 

An initial energy value of 1 Joule (J) is offered equally to all implanted sensors. The number of relay nodes is limited to two with coordinates (1.65, 0.75) and (0.9, 1.65) in accordance with [19]. The packet length is set to 2000 bits, since this is the maximum payload regulated by the IEEE 802.15.6 standard [34]. Table 1 summarizes the simulation parameters. Simulations are conducted in MATLAB and compare the performance of the proposed protocol with our previously proposed WBAN routing protocols: two-relay-based, and incremental-relay-based routing protocols. Other simulation tools such as NS2, NS3, OMNET, and OPNET are also applicable; detailed information can be found in [35,36]. 

**Figure 4:**
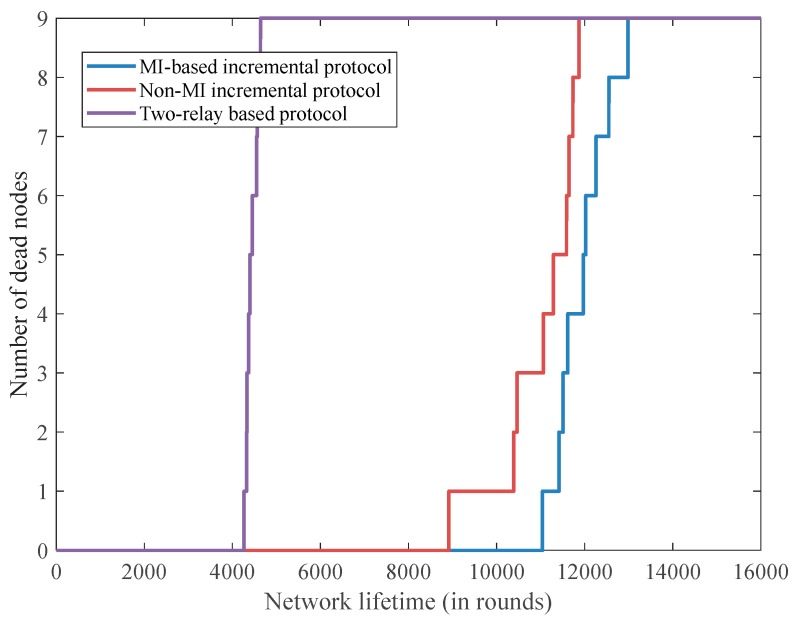
The number of dead implanted sensors versus network lifetime.

It can be seen in Figure 4 that the stability period in the MI-based incremental relaying protocol is around 11,000 rounds, whereas it is 8700 rounds for the non-MI incremental relaying protocol and 4100 rounds for the two-relay-based routing technique. Also, the MI-based incremental relaying protocol achieves the most extended network lifetime at about 12,800 rounds while this is 11,800 rounds for the non-MI incremental relaying protocol and only 4250 rounds for two-relay-based routing. The reasons for the results are as follows: the incremental relaying technique promises longer network lifetime than the static two-relay-based routing scheme because the proposed relay node selection method is capable of balancing the implanted sensors’ energy consumption in each round by considering both transmission distance and the residual energy status of each implanted sensor. Moreover, the MI-based routing protocol allows implanted sensors to not transfer redundant data, which results in extending the stability period and network lifetime.

**Figure 5:**
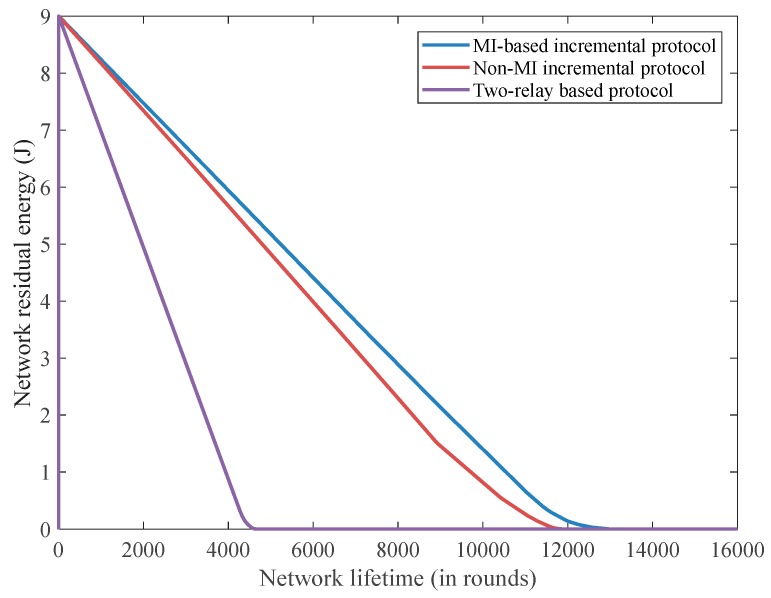
The residual network energy versus the network lifetime.

Figure 5 demonstrates the residual network energy status in each round of the three advanced communication techniques. The MI-based routing technique has the least average energy consumption at around 0.7 mJ per round in comparison to the two-relay-based protocol scheme at 2.1 mJ and the non-MI incremental relaying approach at 0.76 mJ. The MI-based protocol scheme is capable of decreasing redundant power consumption by prohibiting continuously sensed normal and two-relay-based range data to be transmitted, whereas the incremental relaying protocol routing technique needs to send all sensed data and therefore contributes to higher energy waste.

**Figure 6:**
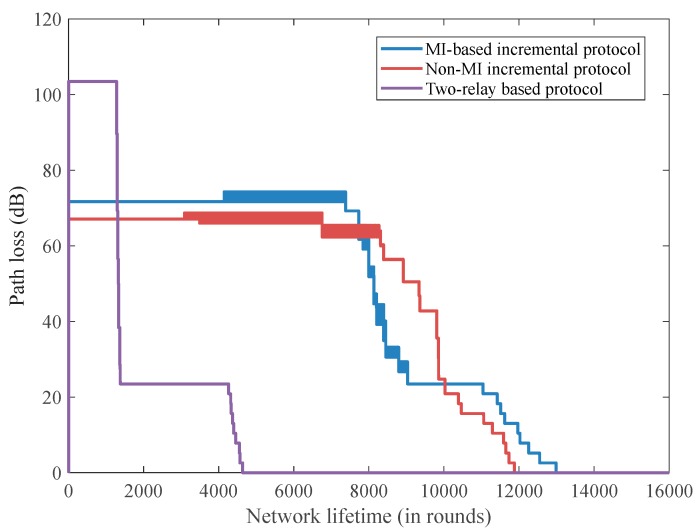
The path loss performance versus the network lifetime.

PL is a crucial parameter in studying the signal power attenuation when transmitting from the transmitter to the receiver. Figure 6 illustrates the PL performance versus the network lifetime of the three proposed protocols. In this paper, the PL exponent *n* of 3.6 and the standard deviation σ of 2.93 are employed as reported in [10]. According to Equation (1), the PL value depends on the transmission distance *d*; the incremental relaying protocols can significantly reduce the overall communication distance, and consequently achieve a smaller PL value than does the two-relay-based routing scheme. Moreover, the PL of the two-relay-based routing protocol decreased significantly since the number of live implanted sensors is reducing rapidly. The non-MI incremental relaying technique achieves smaller PL values than does the MI-based routing protocol for the first 8000 rounds. Then, the instantaneous PL value of the non-MI incremental relaying technique becomes larger than that of the MI-based routing technique. This is because numerous implanted sensors deplete their power and the live transmitting implanted sensors are far away from the relay node or the coordinator for the non-MI incremental relaying technique, while none of the implanted sensors are dead after almost 11,000 rounds in the MI-based incremental relaying method.

**Figure 7:**
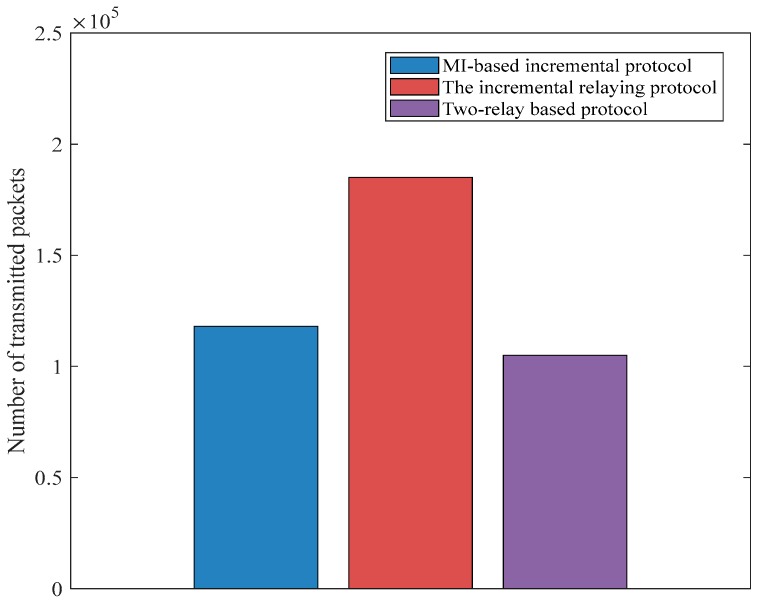
A comparison of three protocols regarding the number of transmitted packets.

We employ the random uniform model for a packet drop probability of 0.3 for the two-relay-based and incremental relaying protocols. As reported in [13], the number of transmitted information packets depends heavily on the number of live implanted sensors. It can be seen from the Figure 7 that the two-relay-based protocol achieves the smallest amount of transmitted packets at approximately 1.05 × 10^5^. This is due to the two-relay-based protocol having a shorter network lifetime as compared with incremental relaying techniques. Moreover, the non-MI-based routing protocol delivers more data packets than the MI-based incremental relaying protocol; this is because MI-based prohibits redundant data transmission when compared with the non-MI incremental relaying technique at the same simulation rounds.

Table 2 summarizes the results of the three types of communication protocol approaches. In a similar way to the results in [19], the two-relay-based communication protocol supports rapid data transmission among all three proposed techniques, which makes it a promising candidate for emergency medical data routing services and applications. The non-MI incremental relaying technique realizes the largest number of data packets transmitted, making it a capable technique for continuous time-varying physiological data monitoring such as electromyogram (EMG) signals. It can be seen that the MI-based routing protocol achieves the best performance in terms of network lifetime compared with the other two communication protocol schemes reported in [10]. In this paper, the proposed communication technique is able to avoid redundant normal range data transmission by employing the algorithm demonstrated in Section 4, whereas the non-MI incremental relaying technique proposed in [10] allows all sensed data transmission, and thus incurs an energy penalty and is not applicable for long-term monitoring services for future rehabilitation monitoring services. Moreover, by investigating the communication link establishment criteria (depending on the predetermined MI threshold), a communication link is only available when the MI value becomes smaller than the predetermined threshold. Unlike the non-MI relaying scheme [10], the MI-based technique allows both a direct communication link and cooperative relaying between the implanted nodes and the coordinator. It is thus capable of supporting real-time patient monitoring services under emergency conditions. Moreover, a direct communication link promises lower propagation delay as compared with the incremental relaying method because data transmission does not need to be received and processed by the corresponding relay node. Future emerging implanted monitoring services can be designed by adopting the appropriate MI predetermined threshold value.

## 7. Conclusions

Network lifetime maximization of wireless in-body area networks is one of the critical challenges in WBANs. In this paper, an MI-based incremental relaying communication protocol was proposed. The network topology is as follows: nine implanted sensors are located inside the human body while relay nodes and the coordinator are attached to a patient’s clothes. Once the MI value is smaller than the predetermined threshold, data transmission is allowed. A minimal transmission length algorithm is utilized to decrease the network energy consumption. Considering the maximization of the network lifetime, various selected QoS mathematical models and the related subjective functions were derived. The results justify that the introduction of the MI-based incremental relaying technique decreases the average energy consumption per bit to around 0.7 mJ which yields a network lifetime extension as compared with the non-MI-based incremental relaying method at approximately 0.76 mJ and the two-relay-based routing scheme at 2.1 mJ.

Our future work is focused on QoS provisioning for mobility support WBAN protocol design on a real experimental testbed such as Delsys EMG sensor networks [36]. Moreover, new networking technology for WBANs is also taken into consideration.

## Figures and Tables

**Table 1:** Simulation parameters.

Parameter	Value (unit)
Frequency	2.4 GHz
$E_{Tx\_elec}$	16.7 nJ/bit
$E_{Rx\_elec}$	36.1 nJ/bit
$E_{amp}$	1.97 nJ/bit
$E_{pro}$	0.3064 nJ/bit
$E_{sen}$	0.12 × 10^−9^ nJ/bit
PL exponent	3.6
PL standard deviation	2.93
$PL_{dB}\left(d_{ref}\right)$	23.49 dB
Payload	2000 bits
Implant sensor initial power $E_{0}$	1 J
Number of implanted sensors	9
$I\left(D_{i};D_{j}\right)$ threshold	0.5
Predetermined BER	10^−3^
Location of implanted sensors	shown in Figure 3b
Location of relays	(1.65, 0.75) and (0.9, 1.65)
$P_{t}$	25 μW

**Table 2:** Simulation results of the proposed three communication protocols.

Protocol Type	Two-Relay-Based Protocol	Non-MI Incremental Protocol	MI-Based Incremental Protocol
Stability period	4100 rounds	8100 rounds	11,000 rounds
Network lifetime	4250 rounds	11,800 rounds	12,800 rounds
Average consumption (per round)	2.1 mJ	0.76 mJ	0.7 mJ
Transmitted packets	1.05 × 10^5^	1.85 × 10^5^	1.18 × 10^5^
